# Gender differences in pain perception among burning mouth syndrome patients: a cross-sectional study of 242 men and 242 women

**DOI:** 10.1038/s41598-024-53074-4

**Published:** 2024-02-09

**Authors:** Elena Calabria, Federica Canfora, Stefania Leuci, Noemi Coppola, Giuseppe Pecoraro, Amerigo Giudice, Alessandro Antonelli, Massimo Aria, Luca D’Aniello, Michele Davide Mignogna, Daniela Adamo

**Affiliations:** 1 Department of Health Sciences, School of Dentistry, University Magna Graecia of Catanzaro, Catanzaro, Italy; 2 Department of Neuroscience, Reproductive Sciences and Dentistry, University of Naples Federico II, Via Pansini N°5, 80131 Naples, Italy; 3 Department of Economics and Statistics, University of Naples Federico II, Naples, Italy; 4 Department of Social Sciences, University of Naples Federico II, Naples, Italy

**Keywords:** Neuroscience, Medical research, Neurology

## Abstract

Several orofacial painful conditions are influenced by gender-related factors, but no studies are available with regard to Burning Mouth Syndrome (BMS). The present study aimed at investigating gender differences among BMS patients and their influence on pain perception. 242 BMS males (BMSm) and 242 BMS females (BMSf) matched for age were consecutively enrolled. Sociodemographic and clinical characteristics were recorded and the numeric rating scale (NRS), the Total Pain Rating Index (T-PRI), the Hamilton rating scale for anxiety and depression (HAM-A, HAM-D), the Pittsburgh sleep quality index (PSQI) and the Epworth sleepiness scale (ESS) were administered. The BMSm presented statistically significant higher levels of education and rate of employment compared to the BMSf (*p*-values: 0.001**). Moreover, the BMSm were greater consumers of alcohol and had a higher BMI than the BMSf (*p*-values: < 0.001**, 0.034*). With respect to systemic comorbidities, cardiovascular diseases were statistically more prevalent among the BMSm, while hypothyroidism was more frequent in the BMSf (*p*-vales: < 0.001**). No differences were noted between the two groups in terms of oral symptoms and in the median scores of NRS, T-PRI, HAM-A, HAM-D, PSQI and ESS. Interestingly, the multivariate regression analysis revealed that, while anxiety, high BMI, poor sleep and high level of T-PRI were correlated to the intensity of pain (NRS) in both groups, low education was additional predictor of pain in BMSf. Further, depression, alcohol and intensity of pain were factors positively associated to the quality of pain (T-PRI) in the BMSm, whereas low education, non-married status and NRS were correlated to the T-PRI, in the BMSf. Surprisingly, smoking was inversely correlated to the intensity of pain and quality of pain respectively in BMSf and BMSm. Sociodemographic and risk factors were found to differently influence pain perception in BMSm and BMSf. Therefore, clinicians should take into account gender differences in the assessment of BMS patients to better tailor the overall pain management.

## Introduction

Burning Mouth Syndrome (BMS) is a chronic neuropathic oral pain disorder characterized by a burning or dysesthetic sensation in the oral mucosa without specific lesions or laboratory findings. BMS typically presents as bilateral oral burning sensations with fluctuating intensity, lasting more than two hours per day for at least three months. Patients frequently report additional discomforting symptoms such as xerostomia (dry mouth), taste disturbances, foreign body sensation in the mouth, and itching or tingling sensations^1^. The prevalence of BMS ranges from 0.7 to 4.6%, however it peaks up to 18% in postmenopausal women^2^.

The pathogenesis of BMS remains largely debated, but it is believed to be multifactorial. Specifically, psychological factors, dysfunctions in the central nervous system, and peripheral small fiber neuropathy are among the most relevant factors thought to contribute to the development and maintenance of BMS^3^.

Notably, recent studies have also highlighted the correlation between BMS and cognitive impairment, as well as the presence of white matter hyperintensities in affected patients, providing additional insights into potential factors associated with its pathogenesis^4,5^.

BMS, as other chronic pain condition, is recognized to be influenced by various factors, including psychological, sociocultural, and biological aspects. Indeed, psychological factors such as depression, anxiety, poor sleep, and adverse social conditions can contribute to the development of chronic pain which in turn can also aggravate the general quality of life of the patients^6,7^. In addition, sociocultural factors like low levels of education and socioeconomic status are deeply linked with a worsening of chronic pain. Of note, there are many biological factors including genetics, age, sex, hormones which play a role in the predisposition and development of chronic pain^8^. Among these, gender has become a focal point for research in the context of chronic pain, primarily because it has been observed that women have a higher prevalence of several chronic pain conditions, for instance fibromyalgia, migraine, chronic tension-type headache, irritable bowel syndrome, temporomandibular disorders, interstitial cystitis, and BMS^2–9^. Extensive research has been dedicated to investigating the role of gender in pain modulation and interpretation, as well as examining gender-related factors that can influence pain perception^10,11^. This line of inquiry aims at understanding how gender influences the way individuals experience and respond to pain.

Indeed, gender differences in pain perception and consequent behavior have been observed, with men being less likely to report or experience chronic pain compared to women. Additionally, females are more likely to report pain in multiple sites than males and tend to use maladaptive coping strategies, which may predispose them to chronic pain and poorer functional ability. Women have generally lower pain thresholds and tolerance and experience greater pain intensity and unpleasantness than men, they also exhibit different sensitivities to analgesia and are more likely to seek treatment for their pain. Studies have shown that women seeking treatment for chronic pain report higher pain intensity and pain-related disability compared to men. In this scenario, the role of estrogens and genetics, including sex-specific differences in pain-related genes, may contribute to these gender-specific differences in pain perception and prevalence^12,13^.

Gender differences have been noted in various chronic orofacial pain conditions, including temporomandibular disorders (TMD). Women, in particular, demonstrate higher sensitivity to experimental pain and a greater prevalence of TMD. Additionally, women exhibit increased temporal summation of heat pain (increased perception of pain over time when repetitive painful stimuli are applied) suggesting that women may have enhanced central nociceptive processing, which refers to increased sensitivity and responsiveness of the central nervous system to pain signals^14,15^.

Although several studies have explored the relationship between pain, psychological factors, and other aspects of BMS^4–7^, to the best of our knowledge, none of the previous studies have specifically focused on evaluating gender differences in a sample of BMS patients.

Therefore, the aim of the current study was to examine potential variations in clinical characteristics, pain perception and psychological profile between female and male individuals with BMS, with the goal of gaining a deeper understanding of the impact of gender on BMS.

Our main hypothesis posits that gender may exert differential influences on pain perception, psychological factors, and social profile in individuals with BMS.

Despite BMS being more prevalent among women, in order to minimize any biases associated with an imbalanced patient sample, equal numbers of male and female subjects were included in this study.

Specifically, the primary objective of this study was to examine potential differences in clinical characteristics, psychological profile, and pain perception between females with BMS (BMSf) and males with BMS (BMSm). Additionally, a secondary objective was to identify possible predictors of pain in both BMSf and BMSm, considering factors such as sociodemographic profile (age, employment, and marital status), body mass index (BMI), risk factors (smoking and alcohol use), other systemic comorbidities, drug consumption and psychological factors.

## Methods

### Study design and participants

The research was structured as an observational study and was conducted at the Oral Medicine Department of the University of Naples "Federico II" from March 2017 to December 2020. The study followed the ethical principles outlined in the World Medical Association Declaration of Helsinki and adhered to the guidelines outlined in the Strengthening the Reporting of Observational Studies in Epidemiology (STROBE) for observational studies^16^.

**Figure 1:**
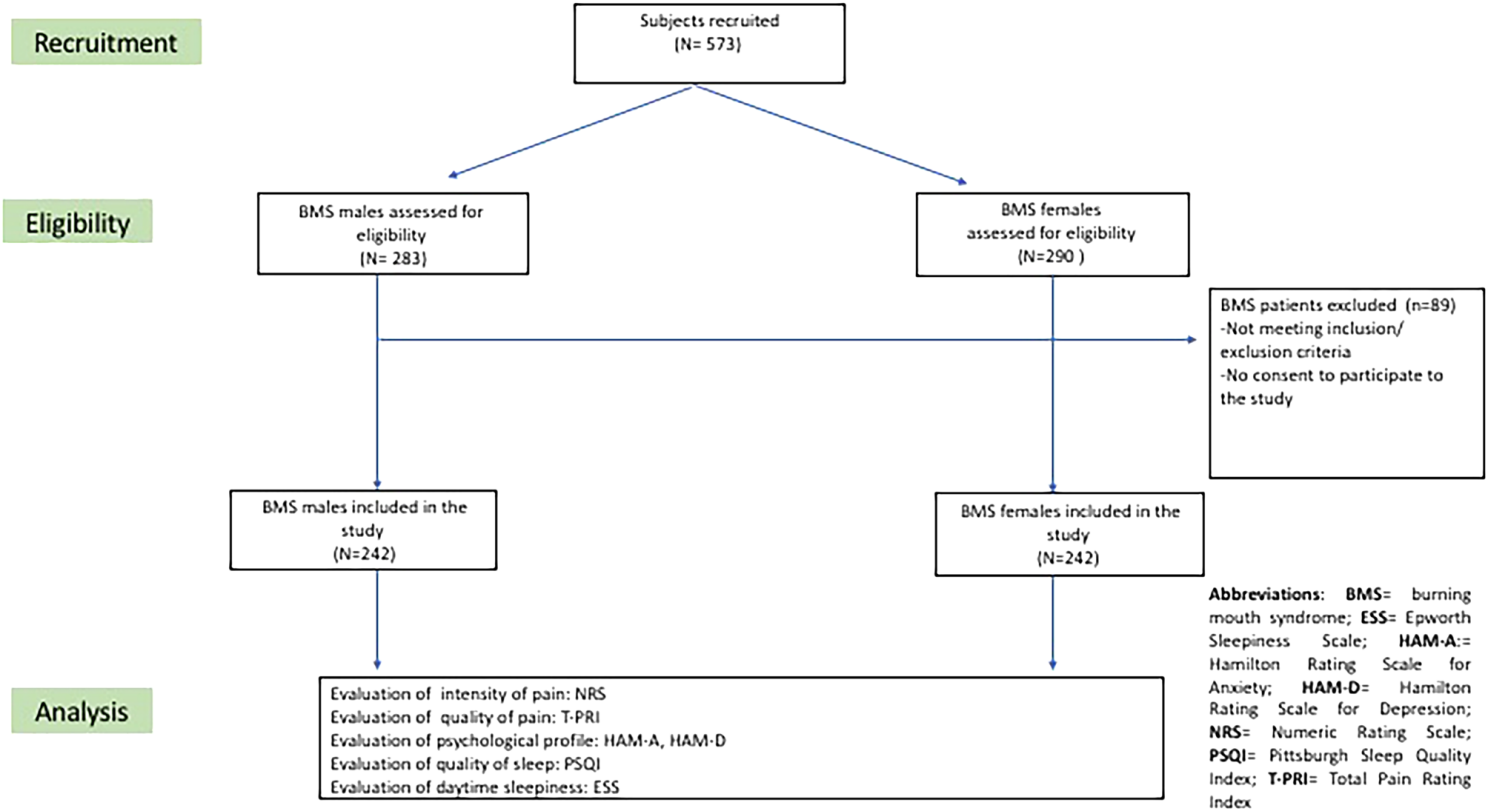
Flow chart of the study.

Consecutive patients seeking initial consultation for BMS were invited to participate in the study. Males and females patients were included in the study and matched based on their mean ages. The recruitment of BMS patients was done through convenience sampling. Initially, a cohort of 573 patients with a confirmed diagnosis of BMS was approached and invited to participate in the study. Subsequently, all individuals were carefully assessed against the predetermined inclusion and exclusion criteria, resulting in the inclusion of a final sample of 484 patients (Fig. 1). Specifically, the inclusion criteria for the BMS group were based on the guidelines provided in the International Classification of Orofacial Pain, 1st edition (ICOP) in 2020^5^. These criteria included:

- Patients experiencing oral burning symptoms lasting for more than 2 h per day, occurring daily, and persisting for more than 3 months, without any clinical mucosal alterations.
- Patients with normal blood test results, including blood count, blood glucose levels, glycated hemoglobin, serum iron, ferritin, and transferrin.
- Patients not receiving treatment with psychotropic drugs.

The exclusion criteria were as follows:

- Patients with diseases that could be identified as causative factors for BMS.
- Pregnant or childbearing patients.
- Patients who were unable to comprehend the questionnaires.
- Patients with a history of psychiatric, neurological, or organic brain disorders.
- Patients with a history of alcohol or substance abuse.
- Patients receiving systemic drugs that could be associated with oral symptoms.
- Patients diagnosed with Obstructive Sleep Apnea Syndrome (OSAS).

### Baseline clinical assessment and data collection

Upon admission, socio-demographic factors were analyzed for each group, including sex, age, years of education, family situation (single, married, divorced, widowed), and employment status (employed, unemployed, retired). Additionally, data on body mass index (BMI)—calculated as weight in kilograms divided by height in meters squared, risk factors such as current smoking status and alcohol consumption, oral symptoms, systemic diseases, and drug consumption were recorded.

A multidisciplinary team comprising two oral medicine specialists (DA and EC) and a board-certified psychiatrist (GP) with at least five years of experience in psychiatric and pain assessment of elderly individuals with chronic orofacial pain evaluated all the patients. Each patient underwent a comprehensive medical analysis, a general medical examination, an intra- and extra-oral clinical examination, and a psychiatric evaluation. During the initial assessment, the psychological profile and complete pain assessment of the patients were conducted. The psychiatrist (GP) in the team performed each patient's evaluation to standardize the clinical procedures.

### Pain, psychological and sleep assessment

Preselected questionnaires, validated in Italian, were administered to each participant. The participants completed these questionnaires, and they were carefully checked to ensure there were no missing data. The following questionnaires were utilized in line with the objectives of the study:

The Numeric Rating Scale (NRS) is a well-validated unidimensional instrument used to measure pain intensity. The score is determined by measuring the distance on the line between "no pain" and the patient's mark, resulting in scores ranging from 0 to 10, where 0 represents no oral symptoms and 10 represents the worst imaginable discomfort^17^.

The Total-Pain Rating Index Questionnaire (T-PRI) is a component of the short form of the McGill Pain Questionnaire (T-PRI) and assesses the quality of pain. The T-PRI is a multidimensional pain questionnaire that measures the sensory, affective, and evaluative aspects of perceived pain. The T-PRI consists of 15 items from the original MPQ, with each item scored from 0 (none) to 3 (severe). The T-PRI score is obtained by summing the item scores, with a range of 0 to 45. Higher scores indicate more severe pain, and there are no established critical cutoff points for interpretation^18^.

The Hamilton Rating Scale for Depression (HAM-D) is a clinician-administered scale used to assess depression. It contains 21 items related to the affective field, and scores can range from 0 to 54. A score greater than 7 indicates impairment, while scores in the range of 7 to 17 indicate mild depression, scores between 18 and 24 indicate moderate depression, and scores over 24 indicate severe depression^19^.

The Hamilton Rating Scale for Anxiety (HAM-A) is a clinician-administered scale used to assess anxiety. It consists of 14 items that measure both psychic anxiety and somatic anxiety. Each item is scored on a scale of 0 to 4, and a total score below 17 indicates mild severity, 18 to 24 indicates mild to moderate severity, and 25 to 30 indicates moderate to severe severity^20^.

Subjective sleep quality and daytime sleepiness were evaluated using the Pittsburgh Sleep Quality Index (PSQI) and the Epworth Sleepiness Scale (ESS), respectively. The PSQI is a self-rated questionnaire that assesses sleep quality and disturbances over a one-month time interval. It generates seven component scores (0–3) related to subjective sleep quality, sleep latency, sleep duration, habitual sleep efficiency, sleep disturbances, use of sleeping medication, and daytime dysfunction. The sum of these scores yields a global score ranging from 0 to 21, with scores above 5 indicating poor sleep quality^21^.

The Epworth Sleepiness Scale (ESS) is a self-administered questionnaire that measures an individual's level of daytime sleepiness. It consists of eight items assessing the propensity for sleep in common situations. Subjects rate their likelihood of dozing in each situation on a scale from 0 (would never doze) to 3 (high chance of dozing). The ESS score is the sum of the eight items, ranging from 0 to 24, with a cutoff value of greater than 10 indicating excessive daytime sleepiness^22^.

### Statistical analyses

The statistical analysis was conducted using SPSS software version 23. Descriptive statistics, such as means, standard deviations, medians, and interquartile range (IQR), were used to analyze the socio-demographic and clinical characteristics of the groups. The Pearson Chi-Square test and Fisher's exact test were employed to assess significant differences between percentages, with *p*-values less than 0.05 or 0.01 indicating moderate or strong significance, respectively.

The non-parametric Mann–Whitney U test was used to compare the median scores of the NRS, T-PRI, HAM-A, HAM-D, PSQI, and ESS between BMSf and BMSm. A *p*-value less than 0.05 was considered statistically significant. The Spearman test was utilized to analyze correlations between qualitative and quantitative predictors and the median scores of NRS and T-PRI in both groups.

Multiple linear regression analyses were performed to identify potential predictors of pain (NRS and T-PRI scores). The regression models considered sociodemographic parameters, psychological profile (HAM-A and HAM-D), and sleep quality (PSQI and ESS). Full models, where all parameters were simultaneously entered, were used to evaluate the relative contributions of these variables to pain. The sample size of 242 patients for each group was determined by fixing a power test value (1-Beta) of no less than 99% and a significance level of no more than 1%. The calculation was conducted using G*Power 3.1.9.7 software from Dusseldorf University. The effect size estimation from a prior research study was used^5^.

### Ethics-approval and consent to participate

The study received approval from the University of Naples “Federico II” Ethical Committee (Approval Number: 251/19, approved on 20th February 2019). Written informed consent to participate in the study was obtained from all enrolled patients.

## Results

A total of 484 BMS patients, 242 BMSf and 242 BMSm matched according to the age (*p*-value: 0.472) met the inclusion criteria and were finally enrolled (Fig. 1). With regard to the sociodemographic characteristics, differences were found with respect to the education, the employment status, alcohol consumption and BMI between the two groups **(**Table 1**).** BMSm presented a statistically significant higher mean of years of education (11.2 ± 4.78) compared to the BMSf (9.74 ± 4.85) (*p*-value: < 0.001**). Further, a statistically significant higher percentage of BMSm were employed (42.1%) or retired (52.1%) in comparison to the BMSf (28.9% and 33.1% respectively), while only the 5.8% of the BMSm were unemployed compared to the 38.0% of the BMSf (*p*-value: < 0.001**), this meaning that overall the rate of patients with a previous or present history of unemployment was much higher among the females. While no differences were detected in terms of smoking habits (*p*-value: 0.373), a statistically significant higher proportion of BMSm were habitual alcohol consumers (42.1%) compared to the BMSf (17.4%) (*p*-value: < 0.001**). Only a moderate statistically significant difference in the distribution of the patients among the BMI categories was detected, with the 42.6% and the 45.9% of the BMSf being normal or overweight against the 35.5% and the 50.4% of the BMSm (*p*-value: 0.034*).

**Table 1:** Socio-demographic profile and risk factors of 242 BMSm and 242 BMSf patients.

Demographic variables	BMSm	BMSf	*p*-value
Age (in years)	Mean ± SD	Mean ± SD	0.472
	64.6 ± 13.3	65.4 ± 9.70	
Education (in years)	Mean ± SD	Mean ± SD	< 0.001**
	11.2 ± 4.78	9.74 ± 4.85	
Family situation	Frequency (%)	Frequency (%)	
Single	24 (9.9)	14 (5.8)	0.402
Married	180 (74.4)	186 (76.9)	
Divorced	13 (5.4)	14 (5.8)	
Widowed	25 (10.3)	28 (11.6)	
Employment	Frequency (%)	Frequency (%)	
Employed	102 (42.1)	70 (28.9)	< 0.001**
Unemployed	14 (5.8)	92 (38)	
Retired	126 (52.1)	80 (33.1)	

Risk factors	Frequency (%)	Frequency (%)	*p*-value
Smoking			
Never	172 (71.1)	185 (76.4)	0.373
< 5 cigarettes	17 (7)	12 (5)	
5–10 cigarettes	10 (4.1)	14 (5.8)	
10–15 cigarettes	18 (7.4)	15 (6.2)	
> 15 cigarettes	25 (10.3)	16 (6.6)	
Alcohol use			
Never	140 (57.9)	200 (82.6)	< 0.001**
Yes (1 unit)	68 (28.1)	33 (13.6)	
Yes (2 units)	25 (10.3)	8 (3.3)	
Yes (> 2)	9 (3.7)	1 (0.4)	
Body Mass Index (kg/m^2^)			
BMI < 18.5	4 (1.7)	2 (0.8)	0.034*
BMI: 18.5–24.9 normal	86 (35.5)	103 (42.6)	
BMI: 25.0–29.9 overweight	122 (50.4)	111 (45.9)	
BMI: 30–34 class I obesity	26 (10.7)	22 (9.1)	
BMI: 35–39.99 class II obesity	3 (1.2)	3 (1.2)	
BMI > 40 class III obesity	1 (0.4)	1 (0.4)	

The significance difference between means was measured by the t-student test.

*Significant 0.01 < *p* ≤ 0.05. **Significant *p* ≤ 0.01.

The significance difference between percentages was measured by the Pearson Chi Square test. * Significant.

0.01 < *p* ≤ 0.05. **Significant *p* ≤ 0.01.

*BMI* Body Mass Index, *BMSf* Burning Mouth Syndrome female patients, *BMSm* Burning Mouth Syndrome male patients. Significant values are in bold.

Table 2 shows the frequencies of the oral symptoms and their localization. In both groups, all the patients complained of a painful sensation described as burning in character. Specifically, pain/burning sensation was diffuse throughout the oral mucosa in 24.4% (59) of the BMSm and in 24.8% (60) of the BMSf, while it was localized in one or more site in the rest of the patients. In both groups, tongue was the most affected site by the pain/burning sensation (49.2% of BMSm and 49.6% of BMSf), followed by the palate, gingiva and lips. Beside the pain/burning, the other most frequent oral symptoms reported in both groups were globus pharyngeus (34.3% of BMSm and 40.9% of BMSm), dysgeusia (14.5% of BMSm and 22.3% of BMSf) and intraoral foreign body sensation (12.8% of BMSm and 13.2% of BMSm). Nevertheless, no statistically significant difference was detected in the frequency distribution of oral symptoms and the oral sites involved.

**Table 2:** Oral symptomatology and pain localization.

Symptoms	BMSm frequency (%)	BMSf frequency (%)	*p*-value
Pain/Burning localized in one or more sites	242 (100)	242 (100)	1.000
Pain/Burning diffuse	57 (23.6)	64 (26.4)	0.529
Xerostomia	32 (13.2)	31 (12.8)	1.000
Dysgeusia	35 (14.5)	54 (22.3)	0.034
Subjective Halitosis	14 (5.8)	21 (8.7)	0.292
Globus pharyngeus	83 (34.3)	99 (40.9)	0.159
Intraoral Foreign Body Sensation	31 (12.8)	32 (13.2)	1.000
Itching	22 (9.1)	16 (6.6)	0.398
Sialorrhea	17 (7)	17 (7)	1.000
Tingling sensation	10 (4.1)	13 (5.4)	0.670
Occlusal Dysesthesia	22 (9.1)	21 (8.7)	1.000
Dysosmia	11 (4.5)	8 (3.3)	0.641
Oral dyskinesia	4 (1.7)	6 (2.5)	0.751

Pain localization	BMSm frequency (%)	BMSf frequency (%)	*p*-value
Gingiva	79 (32.6)	95 (39.3)	0.155
Lips	77 (31.8)	93 (38.4)	0.153
Perioral mucosa	65 (26.9)	76 (31.4)	0.317
Buccal mucosa	64 (26.4)	81 (33.5)	0.112
Tongue	119 (49.2)	120 (49.6)	1.000
Floor of the mouth	61 (25.2)	71 (29.3)	0.358
Palate	75 (31)	96 (39.7)	0.057
Retromolar area	60 (24.8)	76 (31.4)	0.129

The significance difference between the percentages was measured by the Fisher’s exact test.

**Significant with Bonferroni correction 0.004 for the symptoms.

**Significant with Bonferroni correction 0.006 for the sites.

*BMSf* Burning Mouth Syndrome female patients, *BMSm* Burning Mouth Syndrome male patients. Significant values are in bold.

The distributions of systemic comorbidities and drug consumption among the groups is presented in the Table 3. Specifically, a statistically significant higher percentage of the BMSm had a previous history of myocardial infarction (20; 8.3%) compared to the BMSf (4, 1.7%) (*p*-value: > 0.001**), while hypothyroidism was more prevalent among the females (38, 15.7%) compared to the males (13, 5.4%) (*p*-value: < 0.001**), and, as a consequence, also the intake of levothyroxine (*p*-value: 0.001*).

**Table 3:** Frequency of systemic diseases and drug consumption in 242 BMSm and 242 BMSf patients.

Systemic disease	BMSm frequency (%)	BMSf frequency (%)	*p*-value
Systemic comorbidities	178 (73.6)	189 (78.1)	0.288
Hypertension	107 (44.2)	107 (44.2)	1.000
Hypercholesterolemia	78 (32.2)	74 (30.6)	0.769
Previous myocardial infarction	20 (8.3)	4 (1.7)	0.001**
Other cardiovascular diseases	24 (9.9)	17 (7)	0.327
Diabetes type II	25 (10.3)	14 (5.8)	0.094
Diabete type I	10 (4.1)	4 (1.7)	0.173
Lung diseases	8 (3.3)	12 (5)	0.494
Gastrointestinal diseases	37 (15.3)	38 (15.7)	1.000
Endocrine diseases	2 (0.8)	2 (0.8)	1.000
Benign prostatic hypertrophy	34 (14)	0 (0)	< 0.001**
Hypothyroidism	13 (5.4)	38 (15.7)	< 0.001**
Hyperthyroidism	0 (0)	4 (1.7)	0.123
Hepatitis B	2 (0.8)	1 (0.4)	1.000
Hepatitis C	5 (2.1)	5 (2.1)	1.000
Previous malignant disease	14 (5.8)	18 (7.4)	0.584
Neurological disesaes	4 (1.7)	4 (1.7)	1.000
Previous psychiatric hystory	28 (11.6)	46 (19)	0.031
Others	37 (15.3)	53 (21.9)	0.079

Drug consumption	BMSm frequency (%)	BMSf frequency (%)	*p*-value
Systemic medications	143 (59.1)	170 (70.2)	0.013
ACE-inhibitors	35 (14.5)	38 (15.7)	0.800
Calcium Channel blockers	18 (7.4)	19 (7.9)	1.000
Sartans	35 (14.5)	20 (8.3)	0.044
Diuretics	31 (12.8)	23 (9.5)	0.312
Beta‐Adrenergic receptor blockers	36 (14.9)	42 (17.4)	0.537
Statins	57 (23.6)	44 (18.2)	0.179
Metformin	30 (12.4)	17 (7)	0.065
Insulin	4 (1.7)	3 (1.2)	1.000
Antiplatelets	57 (23.6)	43 (17.8)	0.144
Anticoagulants	16 (6.6)	5 (2.1)	0.023
Biphosphonate	0 (0)	9 (3.7)	0.004
Levothyroxine sodium	10 (4.1)	31 (12.8)	0.001**
Proton pump inhibitors	49 (20.2)	43 (17.8)	0.563
Steroids	5 (2.1)	3 (1.2)	0.724
Azathioprine	0 (0)	1 (0.4)	1.000
Other drugs	48 (19.8)	50 (20.7)	0.910

The significance difference between the percentages was measured by the Fisher’s exact test.

**Significant with Bonferroni correction 0.003 for systemic diseases.

**Significant with Bonferroni correction 0.003 for drug consumption.

Abbreviations: *BMSf* Burning Mouth Syndrome female patients, *BMSm* Burning Mouth Syndrome male patients. Significant values are in bold.

Table 4 show the pain assessment, psychological profile and sleep evaluation in the sample.

**Table 4:** Pain assessment psychological profile and sleep in 242 BMSm and 242 BMSf patients.

Clinical parameters	BMSm Median; IQR	BMSf Median; IQR	*p*-value
NRS	6 [0–10]	6 [0–10]	0.943
T-PRI	5 [0–10]	4.5 [0–11]	0.885
HAM-D	12 [4–17]	13 [6–17]	0.149
HAM-A	13 [4–18]	14 [6–18]	0.234
PSQI	8 [4–9]	8 [4–10]	0.445
ESS	6 [4–9]	6 [4–8]	0.975

IQR is the interquartile range. The significance difference between medians was measured by the Mann–Whitney test.

**Significant with Bonferroni correction 0.008.

*BMSf* Burning Mouth Syndrome female patients, *BMSm* Burning Mouth Syndrome male patients, *ESS* Epworth Sleepiness Scale,* HAM-A* Hamilton rating scale for anxiety, *HAM-D* Hamilton rating scale for depression, *NRS* Numeric Rating Scale, *PSQI* Pittsburgh Sleep Quality Index, *T-PRI* Total Pain Rating Index.

There was no difference in the median and interquartile range of the clinical parameters (NRS, T-PRI, HAM-A HAM-D, PSQI, ESS) between BMSm and BMSf but both the BMSm and BMSf presented high median scores of NRS, T-PRI, of HAM-A, HAM-D, PSQI and ESS, confirming that on average BMS patients complain about painful oral symptoms and suffer from anxiety, depression and sleep disorder. Similarly, the distribution of the BMS patients based on the severity categories in relation to NRS, HAM-A, HAM-D, PSQI and ESS did not differ among females and males.

However, symptoms of anxiety and depression as well as sleep disturbances affected the majority of the BMS patients. In details, anxiety and depression (HAM-A and HAM-D scores > 7) were observed in 64% (155) and 61.6% (149) BMSm and in 69.8% (169) and 68.4% (166) BMSf respectively. Further, the 71.1% (172) of BMSm and the 73.1% (177) of BMSf complained of poor quality of sleep (PSQI > 5), while daytime sleepiness was found altered in a small number of patients (11.6% of BMSm and BMSf). With respect to the NRS severity, it is interesting to note that, while all the patients complained about painful symptoms, almost half of them complained of mild pain (NRS 1–5) and the other half of severe pain (NRS > 8) in both group, whereas only few had moderate pain (NRS 6–7) (Table 5).

**Table 5:** Score categories for NRS, T-PRI, HAM-A, HAM-D, PSQI and ESS in 242 BMSm and 242 BMSf patients.

Clinical parameters	BMSm	BMSf	*p*-value
NRS	Frequency (%)	Frequency (%)	
Mild pain 1–5	116 (47.9)	117 (48.3)	0.55
Moderate pain 6–7	13 (5.4)	8 (3.3)	
Severe pain > 8	113 (46.7)	117 (48.3)	
T-PRI	Median; IQR	Median; IQR	
	5 [0–10]	4.5 [0–11]	0.885
HAM-A	Frequency (%)	Frequency (%)	
Normal 0–7	87 (36)	73 (30.2)	0.295
Mild severity 8–17	83 (34.3)	100 (41.3)	
Mild to moderate 18–25	55 (22.7)	57 (23.6)	
Moderate to severe 25–30	17 (7)	12 (5)	
HAM-D	Frequency (%)	Frequency (%)	
Normal 0–7	93 (38.4)	76 (31.4)	0.213
Mild depression 8–16	80 (33.1)	97 (40.1)	
Moderate depression 17–23	58 (24)	53 (21.9)	
Severe depression > 24	11 (4.5)	16 (6.6)	
PSQI	Frequency (%)	Frequency (%)	
PSQI total score < 5	70 (28.9)	65 (26.9)	0.685
PSQI total score ≥ 5	172 (71.1)	177 (73.1)	
ESS	Frequency (%)	Frequency (%)	
Normal range 0–10	214 (88.4)	214 (88.4)	0.837
Mild sleepiness 11–14	26 (10.7)	24 (9.9)	
Moderate sleepiness 15–17	1 (0.4)	1 (0.4)	
Severe sleepiness > 18	1 (0.4)	3 (1.2)	

The significance difference between the percentages was measured by the Fisher’s exact test.

**Significant with Bonferroni correction 0.003. The significance difference between medians was measured by the Mann–Whitney test.

*Significant 0.01 < *p* ≤ 0.05, **Significant *p* ≤ 0.01.

*BMSf* Burning Mouth Syndrome female patients, *BMSm* Burning Mouth Syndrome male patients, *ESS* Epworth Sleepiness Scale, *HAM-A* Hamilton rating scale for anxiety, *HAM-D* Hamilton rating scale for depression, *NRS* Numeric Rating Scale, *PSQI* Pittsburgh Sleep Quality Index, *T-PRI* Total Pain Rating Index.

Tables 6 and 7 show the dependence analyses between the NRS and TPR-I scores and the quantitative variables. In both groups, there was a positive correlation between the NRS and TPR-I scores and the HAM-A, HAM-D, PSQI and ESS, NRS (*p*-values: < 0.001**).

**Table 6:** Linear Correlation analysis between the NRS and T-PRI scores and the quantitative predictors in 242 BMSm patients.

Quantitative predictors	NRS	T-PRI
	ρ (*p*-value)	ρ (*p*-value)
HAM-A	0.714 (< 0.001**)	0.684 (< 0.001**)
HAM-D	0.700 (< 0.001**)	0.708 (< 0.001**)
PSQI	0.525 (< 0.001**)	0.531 (< 0.001**)
ESS	0.285 (< 0.001**)	0.277 (< 0.001**)
NRS	–	0.884 (< 0.001**)

ρ is Spearman’s correlation coefficient. *p*-value—*Moderately significant 0.01 < *p*-value ≤ 0.05; **strongly significant *p*-value ≤ 0.01.

*BMSf* Burning Mouth Syndrome female patients, *BMSm* Burning Mouth Syndrome male patients, *ESS* Epworth Sleepiness Scale, *HAM-A* Hamilton rating scale for anxiety, *HAM-D* Hamilton rating scale for depression, *NRS* Numeric Rating Scale, *PSQI* Pittsburgh Sleep Quality Index, *T-PRI* Total Pain Rating Index. Significant values are in bold.

**Table 7:** Linear Correlation analysis between the NRS and T-PRI scores and the quantitative predictors in 242 BMSf patients.

Quantitative predictors	NRS	T-PRI
	ρ (*p*-value)	ρ (*p*-value)
HAM-A	0.678 (< 0.001**)	0.628 (< 0.001**)
HAM-D	0.662 (< 0.001**)	0.625 (< 0.001**)
PSQI	0.441 (< 0.001**)	0.424 (< 0.001**)
ESS	0.206 (< 0.001**)	0.214 (< 0.001**)
NRS	–	0.856 (< 0.001**)

ρ is Spearman’s correlation coefficient. *Moderately significant 0.01 < *p*-value ≤ 0.05; **strongly significant *p*-value ≤ 0.01.

*BMSf* Burning Mouth Syndrome female patients, *BMSm* Burning Mouth Syndrome male patients, *ESS* Epworth Sleepiness Scale, *HAM-A* Hamilton rating scale for anxiety, *HAM-D* Hamilton rating scale for depression, *NRS* Numeric Rating Scale, *PSQI* Pittsburgh Sleep Quality Index, *T-PRI* Total Pain Rating Index. Significant values are in bold.

The results of the simultaneous multiple linear regression analyses predicting the intensity and the quality of pain (NRS and T-PRI scores) for BMSm and BMSf are represented in Tables 8 and 9 respectively. The first model tested the contribution of the demographic variables, and revealed that the education significantly contributed to the NRS score in both BMSm and BMSf groups (*p*-value: < 0.001*, 0.033 respectively), and to the T-PRI score only in males (*p*-value: 0.001*). Additionally, the employment status and the marital status were found to contribute to the NRS and T-PRI score respectively only in females (*p*-values: 0.045* and 0.036*). The addition of risk factors (smoking habit and alcohol use) and of BMI (model 2) resulted in a significant increase in the R2 value for the NRS and T-PRI in both groups (NRS: DR2 = 7.88%; *p*-value < 0.001** in BMSm, DR2 = 13.75%; *p*-value < 0.001** in BMSf; T-PRI: DR2 = 3.13%; *p*-value < 0.014** in BMSm, DR2 = 8.68%; *p*-value < 0.001** in BMSf). The addition of comorbidities, namely prostatic hypertrophy and myocardial infartation in BMSm and of hypothyroidism in BMSf (model 3) did not contribute to the NRS in either groups, while it resulted in a significant increase in the R2 value for the T-PRI only in females (DR2 = 1.95%; *p*-value < 0.018*). The addition of anxiety and depression (HAM-A and HAM-D) significantly contributed to increase the R2 value of the NRS and T-PRI in females and males (NRS: DR2 = 48.46%; *p*-value < 0.001** in BMSm, DR2 = 43.14%; *p*-value < 0.001** in BMSf; T-PRI: DR2 = 42.60%; *p*-value < 0.001** in BMSm, DR2 = 27.83%; *p*-value < 0.001** in BMSf).

**Table 8:** Multiple linear regression analysis predicting NRS in 242 BMSm and 242 BMSf patients.

NRS Male	Model 1		Model 2		Model 3		Model 4		Model 5		Model 6		Model 7	
	Beta (SE)	*p*-value	Beta (SE)	*p*-value	Beta (SE)	*p*-value	Beta (SE)	*p*-value	Beta (SE)	*p*-value	Beta (SE)	*p*-value	Beta (SE)	*p*-value
Age	− 0.03 (0.03)	0.232	− 0.02 (0.03)	0.516	− 0.04 (0.03)	0.134	− 0.03 (0.02)	0.069	− 0.04 (0.02)	0.101	0.00 (0.02)	0.762	− 0.01 (0.01)	0.716
Years of education	− 0.26 (0.06)	< 0.001**	− 0.26 (0.06)	< 0.001**	− 0.25 (0.06)	< 0.001**	− 0.08 (0.05)	0.100	− 0.17 (0.06)	0.002**	− 0.09 (0.04)	0.018*	− 0.04 (0.03)	0.186
Marital status: Married	0.03 (0.65)	0.958	− 0.12 (0.64)	0.851	0.02 (0.65)	0.971	0.24 (0.46)	0.600	0.20 (0.56)	0.714	0.02 (0.37)	0.954	0.04 (0.33)	0.906
Job: occupied	− 0.95 (0.74)	0.200	− 0.27 (0.72)	0.706	− 0.62 (0.75)	0.404	− 0.99 (0.51)	0.054	− 1.03 (0.63)	0.106	− 0.48 (0.42)	0.251	− 0.32 (0.38)	0.410
Smoker			0.23 (0.65)	0.726									0.63 (0.34)	0.063
Alchool use			− 1.36 (0.61)	0.026									− 0.63 (0.33)	0.058
BMI			0.33 (0.08)	< 0.001**									0.10 (0.04)	0.013*
Benign prostatic hypertrophy					1.36 (0.85)	0.109							0.45 (0.43)	0.299
Previous myocardial infarction					1.99 (1.06)	0.062							0.99 (0.54)	0.067
Anxiety (HAM-A)							0.30 (0.06)	< 0.001**					0.18 (0.04)	< 0.001**
Depression (HAM-D)							0.10 (0.06)	0.096					− 0.05 (0.05)	0.264
Quality of sleep (PSQI)									0.63 (0.08)	< 0.001**			0.12 (0.05)	0.032*
ESS									0.11 (0.08)	0.172			0.00 (0.05)	0.947
T-PRI											0.63 (0.03)	< 0.001**	0.46 (0.03)	< 0.001**
*R*^*2*^ (%)	**7.41**	< 0.001**	**15.29**	< 0.001**	**8.85**	< 0.001**	**55.87**	< 0.001**	**32.53**	< 0.001**	**70.48**	< 0.001**	**77.49**	< 0.001**
*R*^*2*^*change* (%)			**7.88**	< 0.001**	**1.44**	**0.060**	**48.46**	< 0.001**	**25.12**	< 0.001**	**63.07**	< 0.001**	**70.08**	< 0.001**

NRS Female	Model 1		Model 2		Model 3		Model 4		Model 5		Model 6		Model 7	
	Beta (SE)	*p*-value	Beta (SE)	*p*-value	Beta (SE)	*p*-value	Beta (SE)	*p*-value	Beta (SE)	*p*-value	Beta (SE)	*p*-value	Beta (SE)	*p*-value
Age	− 0.07 (0.04)	0.054	− 0.07 (0.03)	0.029*	− 0.07 (0.04)	0.046*	− 0.02 (0.03)	0.368	− 0.06 (0.03)	0.084	− 0.05 (0.02)	0.044*	− 0.03 (0.02)	0.116
Years of education	− 0.16 (0.07)	0.033*	− 0.10 (0.07)	0.128	− 0.16 (0.07)	0.032*	− 0.04 (0.05)	0.436	− 0.15 (0.07)	0.022*	− 0.17 (0.05)	< 0.001**	− 0.09 (0.04)	0.031*
Marital status: Married	− 0.59 (0.70)	0.403	− 0.95 (0.65)	0.146	− 0.58 (0.70)	0.409	− 0.26 (0.52)	0.616	0.01 (0.65)	0.992	0.54 (0.45)	0.234	0.33 (0.39)	0.398
Job: occupied	− 1.62 (0.80)	0.045*	− 1.40 (0.75)	0.064	− 1.60 (0.80)	0.047*	− 0.52 (0.60)	0.392	− 0.96 (0.74)	0.196	− 0.71 (0.52)	0.173	− 0.24 (0.45)	0.590
Smoker			− 3.40 (0.88)	< 0.001**									− 1.29 (0.54)	0.017*
Alchool use			1.03 (0.78)	0.186									0.76 (0.46)	0.097
BMI			0.34 (0.07)	< 0.001**									0.13 (0.04)	0.005**
Hypothyroidism					1.03 (0.81)	0.203							− 0.75 (0.45)	0.096
Anxiety (HAM-A)							0.32 (0.06)	< 0.001**					0.16 (0.04)	< 0.001**
Depression (HAM-D)							0.11 (0.06)	0.067					0.06 (0.04)	0.158
Quality of sleep (PSQI)									0.53 (0.08)	< 0.001**			0.11 (0.05)	0.041*
ESS									0.06 (0.08)	0.500			− 0.09 (0.05)	0.084
T-PRI											0.53 (0.03)	< 0.001**	0.37 (0.03)	< 0.001**
*R*^*2*^ (%)	**4.14**	0.007**	**17.89**	< 0.001**	**4.39**	0.007**	**47.27**	< 0.001**	**19.64**	< 0.001**	**60.24**	< 0.001**	**71.71**	< 0.001**
*R*^*2*^*change* (%)			**13.75**	< 0.001**	**0.26**	**0.203**	**43.14**	< 0.001**	**15.50**	< 0.001**	**56.11**	< 0.001**	**67.58**	< 0.001**

SE are the standard errors of the beta estimates. The *p*-values were obtained from the hypothesis test on regression coefficients.

*Moderately significant .01 < *p*-value ≤ 0.05.**Strongly significant *p*-value ≤ 0.01.

*BMI* Body mass index, *BMSf* Burning Mouth Syndrome female patients, *BMSm* Burning Mouth Syndrome male patients, *ESS* Epworth Sleepiness Scale, *HAM-A* Hamilton rating scale for anxiety, *HAM-D* Hamilton rating scale for depression, *NRS* Numeric Rating Scale, *PSQI* Pittsburgh Sleep Quality Index, *T-PRI* Total Pain Rating Index. Significant values are in bold.

**Table 9:** Multiple linear regression analysis predicting T-PRI in 242 BMSm and 242 BMSf patients.

T-PRI male	Model 1		Model 2		Model 3		Model 4		Model 5		Model 6		Model 7	
	Beta (SE)	*P*-value	Beta (SE)	*P*-value	Beta (SE)	*P*-value	Beta (SE)	*P*-value	Beta (SE)	*P*-value	Beta (SE)	*P*-value	Beta (SE)	*P*-value
Age	− 0.04 (0.04)	0.215	− 0.04 (0.04)	0.269	− 0.05 (0.04)	0.137	− 0.05 (0.03)	0.060	− 0.05 (0.03)	0.136	− 0.01 (0.02)	0.656	− 0.03 (0.02)	0.151
Years of education	− 0.28 (0.08)	0.001**	− 0.28 (0.08)	0.001**	− 0.26 (0.08)	0.002**	− 0.05 (0.06)	0.400	− 0.18 (0.08)	0.022*	0.01 (0.05)	0.899	0.03 (0.05)	0.492
Marital status: Married	0.02 (0.86)	0.980	− 0.32 (0.86)	0.706	0.02 (0.86)	0.977	0.42 (0.65)	0.521	0.21 (0.78)	0.790	− 0.02 (0.48)	0.974	0.03 (0.48)	0.956
Job: occupied	− 0.74 (0.97)	0.445	− 0.12 (0.98)	0.904	− 0.39 (0.98)	0.691	− 0.79 (0.72)	0.274	− 0.79 (0.88)	0.372	0.29 (0.5)	0.603	0.07 (0.55)	0.895
Smoker			− 0.79 (0.88)	0.368									− 1.03 (0.49)	0.036*
Alchool use			− 0.28 (0.82)	0.731									1.52 (0.47)	0.001**
BMI			0.33 (0.11)	0.002**									− 0.05 (0.06)	0.423
Benign prostatic hypertrophy					1.68 (1.12)	0.133							0.42 (0.62)	0.493
Previous myocardial infarction					1.80 (1.39)	0.197							-0.11 (0.78)	0.890
Anxiety (HAM-A)							0.23 (0.08)	0.006**					− 0.03 (0.07)	0.620
Depression (HAM-D)							0.27 (0.09)	0.002**					0.20 (0.07)	0.003**
Quality of sleep (PSQI)									0.71 (0.11)	< 0.001**			− 0.04 (0.08)	0.620
ESS									0.06 (0.11)	0.606			− 0.10 (0.07)	0.145
NRS											1.08 (0.05)	< 0.001**	0.96 (0.07)	< 0.001**
R^2^ (%)	**3.93**	0.008**	**7.07**	< 0.001**	**4.61**	0.008**	**46.53**	< 0.001**	**21.06**	< 0.001**	**69.37**	< 0.001**	**71.82**	< 0.001**
R^2^change (%)			**3.13**	0.014*	**0.68**	**0.161**	**42.60**	< 0.001**	**17.13**	< 0.001**	**65.44**	< 0.001**	**67.89**	< 0.001**

T-PRI female	Model 1		Model 2		Model 3		Model 4		Model 5		Model 6		Model 7	
	Beta (SE)	*P*-value	Beta (SE)	*P*-value	Beta (SE)	*P*-value	Beta (SE)	*P*-value	Beta (SE)	*P*-value	Beta (SE)	*P*-value	Beta (SE)	*P*-value
Age	− 0.04 (0.05)	0.407	− 0.05 (0.05)	0.339	− 0.05 (0.05)	0.340	0.01 (0.05)	0.842	− 0.03 (0.05)	0.569	0.03 (0.03)	0.313	0.03 (0.03)	0.393
Years of education	0.03 (0.11)	0.750	0.09 (0.10)	0.387	0.03 (0.10)	0.759	0.17 (0.09)	0.070	0.03 (0.10)	0.735	0.21 (0.07)	0.003**	0.20 (0.07)	0.005**
Marital status: Married	− 2.13 (1.01)	0.036*	− 2.53 (0.98)	0.010*	− 2.11 (1.00)	0.037*	− 1.76 (0.86)	0.043*	− 1.53 (0.97)	0.116	− 1.48 (0.65)	0.024*	− 1.52 (0.67)	0.024*
Job: occupied	− 1.72 (1.17)	0.142	− 1.48 (1.13)	0.191	− 1.68 (1.16)	0.148	− 0.45 (1.00)	0.651	− 1.04 (1.12)	0.352	0.08 (0.76)	0.914	0.01 (0.77)	0.993
Smoker			− 3.11 (1.32)	0.020*									0.75 (0.93)	0.422
Alchool use			0.52 (1.16)	0.655									− 0.52 (0.79)	0.514
BMI			0.45 (0.11)	< 0.001**									0.06 (0.08)	0.448
Hypothyroidism					2.79 (1.17)	0.018*							1.50 (0.77)	0.053
Anxiety (HAM-A)							0.36 (0.10)	< 0.001**					0.00 (0.08)	0.980
Depression (HAM-D)							0.13 (0.10)	0.180					0.01 (0.07)	0.911
Quality of sleep (PSQI)									0.56 (0.13)	< 0.001**			− 0.03 (0.09)	0.780
ESS									0.19 (0.13)	0.130			0.11 (0.09)	0.188
NRS											1.11 (0.06)	< 0.001**	1.08 (0.09)	< 0.001**
R^2^ (%)	**1.01**	**0.165**	**9.69**	< 0.001**	**2.96**	0.031*	**28.84**	< 0.001**	**11.34**	< 0.001**	**58.95**	< 0.001**	**58.96**	< 0.001**
R^2^change (%)			**8.68**	< 0.001**	**1.95**	0.018*	**27.83**	< 0.001**	**10.32**	< 0.001**	**57.93**	< 0.001**	**57.95**	< 0.001**

SE are the standard errors of the beta estimates. The *p*-values were obtained from the hypothesis test on regression coefficients.

*Moderately significant .01 < *p*-value ≤ .05.**Strongly significant *p*-value ≤ .01.

*BMI* Body mass index, *BMSf* Burning Mouth Syndrome female patients, *BMSm* Burning Mouth Syndrome male patients, *ESS* Epworth Sleepiness Scale, *HAM-A* Hamilton rating scale for anxiety, *HAM-D* Hamilton rating scale for depression, *NRS* Numeric Rating Scale, *PSQI* Pittsburgh Sleep Quality Index, *T-PRI* Total Pain Rating Index. Significant values are in bold.

The addition of quality of sleep (PSQI, ESS) in model 5, resulted in a significant increase in the R2 value for the NRS and T-PRI in both males and females (NRS: DR2 = 25.12%; *p*-value < 0.001** in BMSm, DR2 = 15.50%; *p*-value < 0.001** in BMSf; T-PRI: DR2 = 17.13%; *p*-value < 0.001** in BMSm, DR2 = 10.32%; *p*-value < 0.001** in BMSf).

In Table 8 the addition of the quality of pain (T-PRI) resulted in a significant increase in the R2 value for the NRS in both males and females (NRS: DR2 = 63.07%; *p*-value < 0.001** in BMSm, DR2 = 56.11%; *p*-value < 0.001** in BMSf).

Similarly, in Table 9, the addition of the intensity of pain (T-PRI) also resulted in a significant increase in the R2 value for the NRS in both males and females (DR2 = 65.44%; *p*-value < 0.001** in BMSm, DR2 = 57.93%; *p*-value < 0.001** in BMSf).

The final full model in Table 8, in which all of the variables were entered simultaneously could explain the 70.08% and the 67.58% of the variance in the total NRS score (pain intensity) in BMSm and BMSf respectively. In details, the most contributing factors in both BMSm and BMSf were the BMI, anxiety, quality of sleep and T-PRI (BMSm *p*-values: 0.013, < 0.001, 0.032, < 0.001, < 0.001; BMSf *p*-values: 0.005, < 0.001, 0.041, < 0.001 respectively). In addition, only in females the variable of less years of education was predictive of higher intensity of pain, while the presence of smoking habit was inversely correlated with pain intensity (*p*-values:0.031, 0.017).

Similarly, the final full model in Table 9, in which all of the variables were entered simultaneously could explain the 67.89% and the 57.95% of the variance in the total T-PRI (quality of pain) in BMSm and BMSf respectively. In details, the most contributing factors to quality of pain in BMSm were alcohol use, depression and NRS (*p*-values: 0.036, 0.001, 0.003, < 0.001), whereas the smoking habit, was negatively correlated to T-PRI (*p*-value: 0.036). Differently, predictors were low education, unmarried status and high scores in NRS (*p*-values: 0.005, 0.024, < 0.001) in BMSf.

## Discussion

Gender differences in the prevalence and in the assessment of diseases have long been a subject of scientific investigation and societal interest. It is widely recognized that biological, genetic, hormonal, and behavioral factors can influence disease susceptibility and presentation between males and females. These differences have significant implications for public health, medical research, and the development of targeted healthcare strategies and have given rise to a gender-oriented medicine which has been advocated by the World Health Organization (WHO) since 1998^23^.

In the context of chronic pain, gender-oriented medicine seeks to understand how sex and gender differences can influence the prevalence, presentation, and management of different chronic pain conditions. Some conditions such as fibromyalgia, TMD, and migraines, complex regional pain syndrome and also BMS exhibits a higher prevalence in females compared to males^3,24–26^. Several studies have consistently reported that females show a significantly higher risk of developing BMS, with rates two to three times higher than males, particularly during the post-menopausal phase^3^. Over the recent years, the recognition of this prevalence disparities between genders in BMS and in other chronic pain conditions have prompted research to explore potential factors explaining this imbalance.

The exact mechanisms underlying this gender disparity are not yet fully understood. However, hormonal influences have been suggested as a potential contributing factor in the pathophysiology of BMS and may explain the higher female predisposition^27^. Indeed, during menopause the level of estrogen and progesterone decline significantly, leading to hormonal imbalances. It is believed that this hormonal fluctuation may disrupt normal functioning of nervous system increasing vulnerability of the peripheral nerves in the oral cavity making them more susceptible to damage or dysfunction. Indeed, the lower estrogen levels on one side may lead to an upregulation of transient receptor potential vanilloid 1(TRPV1) and P2X purinoceptor 3 (P2X3) on the other side may contribute to enhanced signaling of nerve growth factor (NGF) and TrkA, leading to increased activity of TRPV1 resulting in an increased sensitivity to pain stimuli. Furthermore, decreased estrogen levels can heighten pain sensitivity by reducing estrogen-dependent modulation of P2X3 in sensory neurons^27^. Additionally, the lower gonadal steroid production inherent to menopause, combined with chronic stress, act on HPA axis leading to increased levels of circulating corticosteroids, particularly cortisol. This overproduction of cortisol has been observed in the saliva of BMS patients, having a detrimental effect on the neural tissue, and may contribute to irreversible neurodegenerative alteration in the central or peripheral nervous system (for instance of the small nerve fibers in the oral mucosa)^28^. Furthermore, progesterone has anti-inflammatory properties, and its decrease during menopause may contribute to inflammation in the oral tissues, further exacerbating BMS symptoms and increasing pain sensitivity^29^.

In this context, the hormonal imbalance may act as a trigger factor in the development of BMS in females. Also, estrogens have mood-stabilizing effects, playing a crucial role in promoting emotional well-being. When estrogen levels decline, particularly during perimenopause and menopause, the risk of developing mood disorders, including depression and anxiety, increases^30^. These mood disorders, in turn, are considered risk factors for the development of BMS^31^. Besides, several studies reported that women generally exhibit a higher tendency to seek healthcare services and report symptoms compared to men^12^. This inclination towards seeking medical assistance could contribute to the observed higher prevalence of BMS in females.

In accordance with previous studies, this recognized disparity in disease prevalence between sex is also observed in our Oral Medicine Unit with the male-to-female ratio of 1:3. Despite this difference, there has been limited research on the impact of gender and related factors on pain perception and psychological profiles in BMS. Therefore, the objective of this study was to gain a deeper understanding of potential gender-related differences in the manifestation of BMS, which is why an equal number of subjects were enrolled in both groups.

In the majority of the studies is overwhelmingly documented that women are more sensitive to pain and less tolerant of pain than men^32^. Specifically, this gender differences in pain perception has been evaluated also in some types of chronic orofacial pain conditions such as TMD and migraines^15,33,34^. These gender differences in pain processing in TMD and migraines patients have been attributed to various factors such as psychosocial and cultural factors but especially, as previously reported, to hormonal fluctuations that may affect pain experience^35^.

Differently in this study, the analysis of the symptomatology revealed no significant differences in the prevalence of burning sensation and additional symptoms between the male and female groups. Similarly, there were no reported variations in the location of symptoms between two groups. Additionally, no significant differences were found in the intensity and quality of pain experienced by male and female patients. This finding indicates that both male and female patients with BMS exhibit similar pattern of symptoms with a lack of gender-based disparities in symptoms’ prevalence, location, intensity, and quality of pain suggesting that the manifestation of BMS is comparable between males and females in terms of the experienced burning sensation and associated symptoms.

These results are in accordance with a meta-analysis published in 2012 where authors report that many of the scores of studies reporting on sex differences in human pain sensitivity in the published literature (122 studies met the inclusion criteria) featured non-statistically significant differences^36^.

To interpret these findings, it is crucial to take into account that around 70% of the patients diagnosed with BMS (Burning Mouth Syndrome) are aged over 65 years. This indicates that the average age of onset for BMS, especially in Italy, has shifted to a few years later, likely due to the increase in life expectancy among individuals. Specifically, in this study, out of the total participants, 166 males (68.6%) and 182 females (75.3%) were older than 65 years of age.

This shift in the age of onset of the disease has significant implications. Indeed, it is known that the hormonal fluctuations typically associated with menopause tend to decrease with the age and have less impact on the symptomatology and psychological profiles in the older individuals with BMS.

The diminishing effect of hormonal changes could potentially account for the lack of significant differences in pain perception observed among our patients, suggesting the presence of shared underlying mechanisms that may contribute to BMS in both genders during old age. Ultimately, the lack of variation in psychological factors or sleep quality may also contribute to explain the absence of pain perception differences between the two groups.

Furthermore, it is worth highlighting that the median age of disease onset in our study samples was approximately 65 years old which is markedly different from the age of onset observed in TMD and migraines, where sex differences in pain perception have been reported^15–26^.

Therefore, the lack of statistically significant differences in pain perception and associated symptoms in our sample of elderly patients further reinforces the concept that with age, hormonal variations tend to diminish in both women and men and may not play a significant role in pain perception.

The equal representation of males and females in the study sample has allowed for a more robust analysis of gender-related differences in BMS suggesting that, despite gender variations in the prevalence of BMS, there are notable similarities in certain aspects of the condition between men and women.

As the onset of BMS in patients has consistently been observed to occur at an older age, there is also a growing need to focus on developing treatment approaches that are specifically tailored to this particular population. This is crucial, given the higher prevalence of systemic diseases associated with the syndrome in older individuals^37,38^. Indeed, a high prevalence of systemic comorbidities in both male and female groups have been found. Specifically, 73.6% of males and 78.1% of females had concurrent systemic diseases, with hypertension and hypercholesterolemia being the most common comorbidities without statistically significant differences in the prevalence of these conditions between the two groups. Consistent with existing literature, we found a statistically significant difference in the prevalence of hypothyroidism between males and females. Indeed, it is known that the influence of female sex hormones and specific life stages unique to women, such as pregnancy and menopause, may affect the thyroid function^39^. Given the potential implications for patients with BMS, it is essential to detect hypothyroidism at an early stage. Left untreated, this condition has been associated with changes in pain perception and sensory abnormalities over time. Indeed, some studies have reported that individuals with hypothyroidism may experience alterations in taste perception^40^ and heightened sensitivity to pain^41^, potentially contributing to the development or exacerbation of BMS symptoms^42^. Therefore, it could be essential identify gender-related comorbidities in the assessment of BMS in order to improve symptom management and over well-being of patients affected.

From the analysis of the psychological profiles, our findings align with previous studies, indicating that a majority of BMS patients suffered from anxiety, depression, and sleep disturbances^43–45^. This analysis did not reveal any significant disparities in the levels of anxiety, depression, or sleep disturbances between male and female BMS patients. This suggests that, within the context of BMS, gender does not appear to influence the prevalence or severity of psychological symptoms.

On the contrary, clinical and preclinical studies have consistently demonstrated sex differences in the neurobiological mechanisms underlying depression and anxiety. These disorders tend to impact women disproportionately, with a higher likelihood of diagnosis^46^. However, considerable evidence supports that the gender disparities on the prevalence of mood disorders decrease in later life^47^.

Therefore, the lack of gender differences in the prevalence of mood disorders in this sample of BMS patients can be attributed to older age of the subjects. The presence of a similar psychological profile, as well as similar pain and symptom experiences, could potentially be attributed to shared pathological brain alterations in both genders. This may particularly involve structural brain connectivity alterations in the regions comprising the pain matrix and medial pain ascending pathway responsible for pain control and modulation and for the emotional-affective profile of BMS^48^. This hypothesis helps to explain the parallel presence of pain-related alterations and mood disturbances, contributing to the occurrence of associated psychiatric comorbidities.

However, despite the similarity in the intensity and quality of pain between both sexes in this study, a noteworthy difference in pain predictors has been identified, suggesting a distinct interpretation and role of pain in the lives of individuals based on their gender. It is known that males and females exhibits some differences in sociodemographic, cultural, risk factor, and mood disorders; similarly, in this study males showed higher levels of education and employment compared to females and had more risk factors such as higher BMI and alcohol consumption.

Through multivariate regression analysis, we found that anxiety, high BMI, poor sleep, and high levels of T-PRI (a pain-related index) were predictive of higher pain levels (measured by NRS) in both male and female BMS patients, while low education was an additional predictor of pain intensity in BMS females only. Further, in terms of the quality of pain, the predictors differed between the genders. In BMS males, depression, alcohol consumption, and pain intensity (NRS) were positively associated with pain quality (T-PRI). Conversely, low education, unmarried status, and pain intensity (NRS) were correlated with pain quality (T-PRI) in BMS females. Interestingly, the smoking habit was inversely correlated to the severity of pain (NRS) and quality of pain (T-PRI) respectively in male and female patients.

Overall, sociodemographic disparities between males and females have an impact on pain perception, particularly in females. Specifically, women tend to have lower education levels compared to men, and this, along with unmarried status, significantly influences pain in females. These findings align with another study on Temporomandibular Disorders (TMD), which conducted a large-scale epidemiological study on TMD patients and demonstrated that lower-educated females were more likely to experience widespread and severe pain complaints^49^. Population studies consistently indicate that the prevalence of chronic pain is inversely related to socioeconomic factors. Individuals who are socioeconomically deprived are not only more likely to experience chronic pain compared to those from more affluent backgrounds, but they also tend to experience more severe pain and higher levels of pain-related disability^50,51^.

Interestingly, smoking emerged as a factor contributing to a less severe pain in both genders. Specifically, it predicted lower scores of NRS in females and lower scores of T-PRI in males. These findings may be in contrast with the existing literature, which consistently suggests that smoking may play a role in the etiopathogenesis of various chronic pain conditions in a dose-dependent manner^52,53^. For instance, a large-scale study involving 3,251 patients with TMD found that smokers reported significantly higher pain severity scores^54^. However, our finding may be explained in light of the effects of nicotine on oral mucosa. Indeed, it is well known that nicotine causes pain and oral irritation by activating the neuronal nicotinic acetylcholine receptors expressed by trigeminal nociceptors which in turns stimulate neurons in the trigeminal subnucleus caudalis (Vc). These neurons in the Vc show excitatory responses when exposed to nicotine applied to the tongue, however exhibit a gradual decrease in firing upon subsequent applications, indicating a desensitization of peripheral sensory neurons. This correlates with the findings of human psychophysical experiments, where individuals reported a progressive decline in ratings of oral irritation^55^. Moreover, there is evidence of the anti-nociceptive effect of nicotine in human studies exhibiting small to moderate increase in pain threshold and pain tolerance^56^.

Another factor contributing to the quality of pain in males was alcohol consumption. The relationship between alcohol and chronic pain is a topic of debate in the literature. Some studies suggest a non-linear inverse association between alcohol consumption and the occurrence of chronic pain^57^. Furthermore, it has been proposed that light and moderate drinkers have a lower risk of chronic pain compared to abstainers and heavy drinkers^58^. However, there is also evidence suggesting that patients may use alcohol as a form of self-medication for chronic pain, as alcohol has analgesic properties. Experimental studies indicate a potential causal relationship between pain and alcohol intake, particularly among males who drink to alleviate their pain^59,60^.

Finally, both female and male BMS patients exhibited similar predictive factors for pain intensity (NRS), including anxiety and poor sleep quality, which are known to contribute to increased pain. However, depression emerged as a predictor exclusively in males, specifically associated with the quality of pain. This finding may also be linked to the observed alcohol misuse reported by depressed males. These gender-specific associations shed light on the complex interplay between psychological factors and pain experience in BMS patients^61^.

In light of these findings, it emerges that a comprehensive understanding of the multifactorial nature of BMS, encompassing both biological and socio-cultural and psychosocial aspects, is crucial for delivering targeted and effective treatments to male and female patients alike. A holistic evaluation necessitates the assimilation of comprehensive data on education, employment status, BMI, alcohol consumption, and smoking patterns for all BMS patients. In consideration of this, a multidisciplinary paradigm, encompassing experts such as nutritionists, psychologists, and physical therapists, becomes indispensable. This collective expertise can offer a nuanced approach that not only addresses the clinical symptoms but also delves into the sociodemographic determinants in BMS management, aiming for optimal patient-centered outcomes. It is imperative to devise educational strategies, with an emphasis on assisting females of lower educational backgrounds, to deepen their comprehension of BMS and empower them with effective symptom management tools. Furthermore, specialized counseling sessions targeting distinct coping mechanisms should be extended to specific subsets, notably unmarried females and males exhibiting signs of alcohol consumption or depression.

The results of the present research should be considered in light of some limitations. Firstly, certain social support factors, including patient income, accessibility to the healthcare system, and the presence of family support, were not considered in the study. Additionally, hormonal evaluations were not conducted for both genders, which could have provided valuable insights.

In this study, we focused our evaluation solely on anxiety, depression, and sleep disturbances, overlooking other psychiatric conditions more commonly associated with men, such as substance use disorders, antisocial personality disorder, and certain types of impulse control disorders. These conditions may potentially play a significant role in pain perception among male patients.

Furthermore, it is worth noting that the recruitment of participants was limited to tertiary referral Oral Medicine Units, which may not fully represent the entire population of individuals affected by BMS.

## Conclusion

This research sheds light on the complex interplay of various factors in the manifestation and experiences of BMS across genders.

The clinical manifestations, pain severity and the psychological profiles not significantly differ between male and female BMS patients this possibly suggesting potential shared underlying pathogenic mechanisms, especially in elderly patients where hormonal fluctuations may diminish. However, sociodemographic factors, including education levels, employment, BMI, and alcohol consumption, may influence pain perception in both genders. Lower education and unmarried status are associated with increased pain severity in females, while alcohol consumption and depression to a poorer quality of pain in males. Smoking, contrary to expectations, is inversely correlated with pain severity in females and intensity of pain in males.

These insights pave the way for the development of tailored lifestyle modification programs, addressing specific gender-related needs in BMS management. Such programs could focus on mitigating factors like BMI, alcohol consumption, and smoking habits, considering their established connection with pain perception.

Future research is needed to further investigate the complex relationship between education, employment, and lifestyle in modulating pain experiences by gender, and to explore hormonal fluctuations in males and females in order to address the existing gaps in knowledge surrounding gender differences in BMS.

## Data Availability

The datasets used and/or analysed during the current study are available from the corresponding author on reasonable request.

## Abbreviations

BMSf: Burning mouth syndrome female patients
BMSm: Burning mouth syndrome male patients
COFP: Chronic orofacial pain
ESS: Epworth Sleepiness Scale
HAM-A: Hamilton Rating Scale for Anxiety
HAM-D: Hamilton Rating Scale for Depression
NRS: Numeric Rating Scale
PSQI: Pittsburgh Sleep Quality Index
TMD: Temporomandibular Disorders
T-PRI: Total Pain Rating Index
